# The Bedding and Linen Department—VII

**Published:** 1908-06-13

**Authors:** 


### NURSING ADMINISTRATION.

### THE BEDDING AND LINEN DEPARTMENT.

### VII.—MAKING AND MENDING.

THE strength of the linen department lies in its reserves. If the matron has to go buying on the occasion of the annual cleaning, when ward supplies are made up to stock, she will be overburdened with anxieties, and in all probability the work of choosing and making will be so hastily performed, that many pounds will be wasted. To keep the linen on a solid and dignified basis, specifications should be made out once or twice a year sufficient to meet all possible demands on the stock, and when the department is once in working order, economy will be effected from the mere fact of being able always to proceed with deliberation. There exists great difference of opinion and practice in regard to the price and quality of the more ordinary materials needed in institutions. We quote a specification for articles required in a clinical hospital of considerable size.

SPECIFICATION.

| Linen | At per Yard s. | d. | Amount Required |
|---|---|---|---|
| 1. Sheeting, ward (60 in.)... ... | 1 | 1¼ | 4,500 yds. |
| 2. Sheeting, sisters (60 in.) ... | 1 | 6 | 100 ,, |
| 3. Sheeting, draw (36 in.) ... | | 8½ | 1,500 ,, |
| 4. Table linen, ward (6 × 4) ... | 1 | 7½ | 200 ,, |
| 5. Pillow, linen, ward (42 in.) ... | | 10½ | 300 ,, |
| 6. Pillow, linen, sisters (42 in.) ... | 1 | 1 | 100 ,, |
| 7. Locker cloth (27 in.) ... ... | | 6¼ | 500 ,, |
| 8. Flax (36 in.) ... ... ... | | 6¾ | 1,000 ,, |
| 9. Towelling, huckaback (27 in.).. | | 7¾ | 500 ,, |
| 10. Towelling, honeycomb (27 in.).. | | 5½ | 1,000 ,, |
| 11. Towelling, round crash (18 in.) | | 4¾ | 1,000 ,, |
| 12. Towelling, nurses' bath ... | | 8¾ | 100 towels |
| 13. Towels, blue typed (nurses) ... | | 9¼ | 100 ,, |
| 14. Croft flannel (22 in.) ... ... | | 4½ | 2,000 yds. |
| 15. Flannelette, white (32 in.) ... | | 3¼ | 100 ,, |
| 16. Check dusters ... ... ... | 2 | 3 doz. | 12 doz. |
| 17. Calender cloth (114 in.) ... | 1 | 8¼ | 200 yds. |
| 18. Calender felt (118 in.), for cutting into lengths of 16 ft. 6 in.) | 9 | 6 | 99 ,, |

The process of making up the linen, even when it means no more than cutting into lengths and hemming at both ends, is very heavy work. In very small institutions, where no extra labour is available for this work, the linen is sometimes bought ready made. But it is a mistake, even in a cottage hospital, to have no one whose special business it is to do work of this kind. The matron ought, at the very least, to have at her disposition some respectable outside worker, paid by the week to undertake the charge of the linen, under her own superintendence, and the cost of her modest wages would speedily be saved out of the extra cost devoted to buying ready-made articles. In a hospital of any size, the linen-room staff sometimes constitutes a difficulty because of want of space. In most cases it is wiser to have one good needle-woman in the house, whose business it is to superintend, cut out, and teach subordinates, and be generally responsible to the matron, housekeeper, or sister, in charge of this department. Outside workers can then be added as required, and the work will be most acceptable to women, who from lameness or other inability, are cut off from ordinary day labour. But the matron will probably not find good needlewomen ready to her hand, and must be prepared as usual to train the staff. Low wages for a beginning, with an increase as proficiency is acquired in machining, etc., will stimulate the apprentices, and give them that bracing sensation of being gradually turned into skilled workers which ought to run right through the hospital.

Much discomfort will be avoided, if the business of marking is intelligently done. The name of the hospital should be legibly marked on the top and bottom of each article, and for this, Cash's woven marks are very suitable; some matrons mark the middle of their sheets also, but if people want to steal sheets, it is not an extra mark which will deter them. The end figure of the year is a useful addition which will serve to throw light on the wearing qualities of the linen in use. In addition to this general mark, there should be a detachable mark, to show what ward or department of the hospital this special article belongs to. The ward marks may be merely a letter of the alphabet, and in this case each article can be boldly marked by hand in the linen room. Marking ink is seldom entirely satisfactory. A linen register should be kept up in the stock room, independently of the Hospital Linen Register, of which we have already given a specimen page. The hospital linen register contains no note of new purchases, and is simply concerned to show what is used in each different department, and what is condemned. The stock room linen register indicates the exact quantity of material bought, the old articles turned to account, the number of articles made, or re-made, the number taken into use and thus transferred to the hospital linen register, and the number of articles remaining in stock when these transfers have been made.

STOCK-ROOM LINEN REGISTER.

*Half-year ending*

| Articles | New Material | Number Made | Number Taken into Use | Number in Stock |
|---|---|---|---|---|
| Sheets ... ... | 50 yards | 25 | 22 | 20 |
| Draw-sheets ... | (Disused sheets) | 75 | 83 | 45 |
| Pillow linen, etc. etc. ... | 48 yards | 60 | 50 | 23 |

Let no one think that the keeping of these registers is waste of time. It is by such exact observance of routine that time is saved and money accounted for, in this perplexing department of hospital economy.

